# Supplementary material for: Dietary Intake of Rosmarinic Acid Increases Serum Inhibitory Activity in Amyloid A Aggregation and Suppresses Deposition in the Organs of Mice
Source: Int J Mol Sci. 2020 Aug 21;21(17):6031. doi: 10.3390/ijms21176031 (PMC7504104; doi:10.3390/ijms21176031)
Supplement: Supplementary file 1 [file ijms-21-06031-s001.pdf]

International Journal of
Molecular Sciences

Supplementary Materials

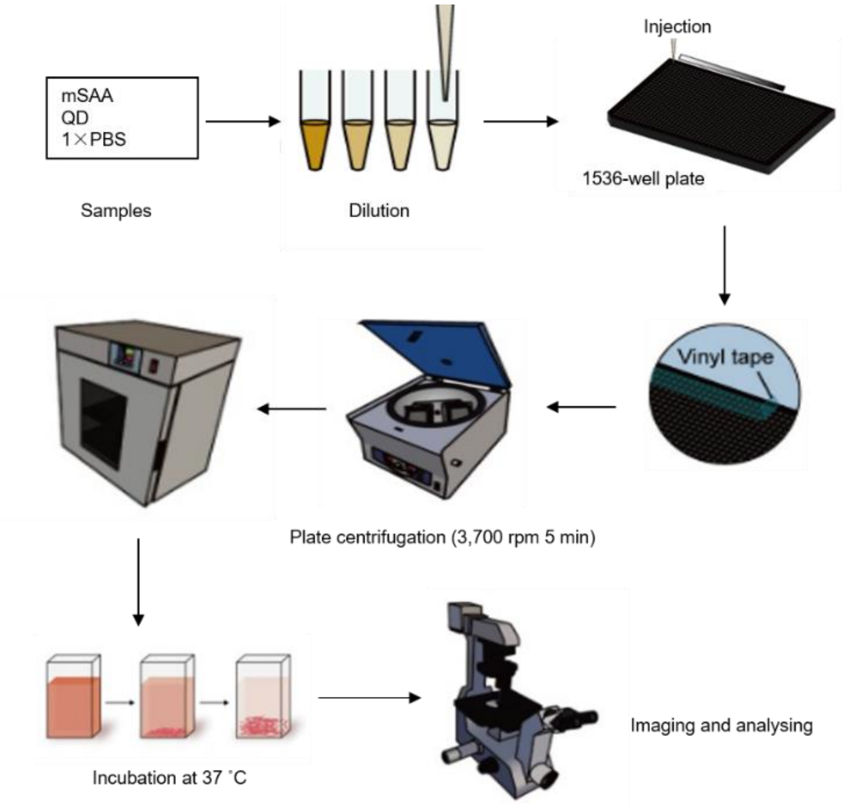

Figure S1. A flow diagram of the microliter-scale high-throughput screening system. Amyloid proteins are mixed with various concentration of inhibitors in a 1536-well plate. After incubation, they are imaged by fluorescence microscopy and the EC_{50} is calculated from fluorescence images.

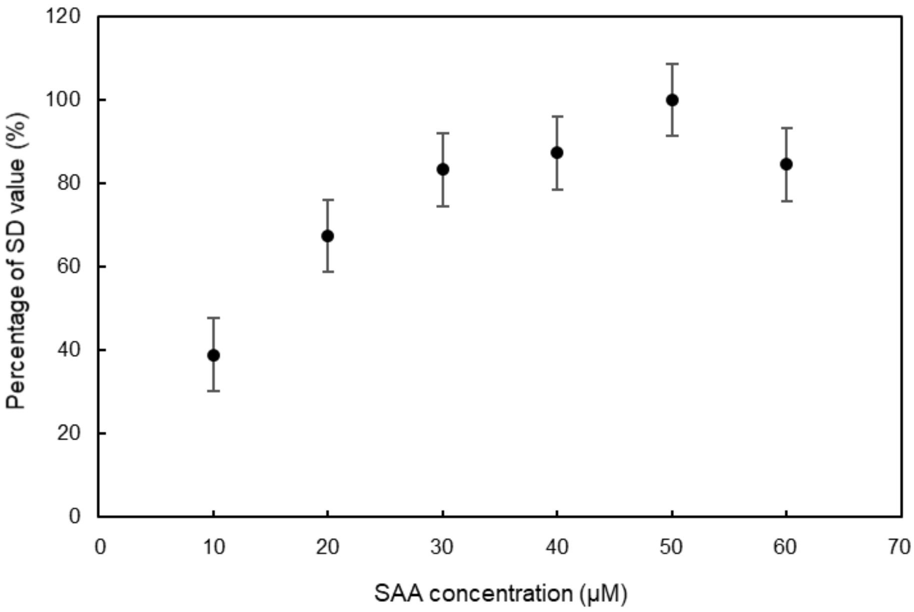

Figure S2. Determination of the optimal SAA protein concentration. SAA protein at approximately 50 μM in an in vitro experiment showed a maximum SD value in the MSTHS system with QDs.

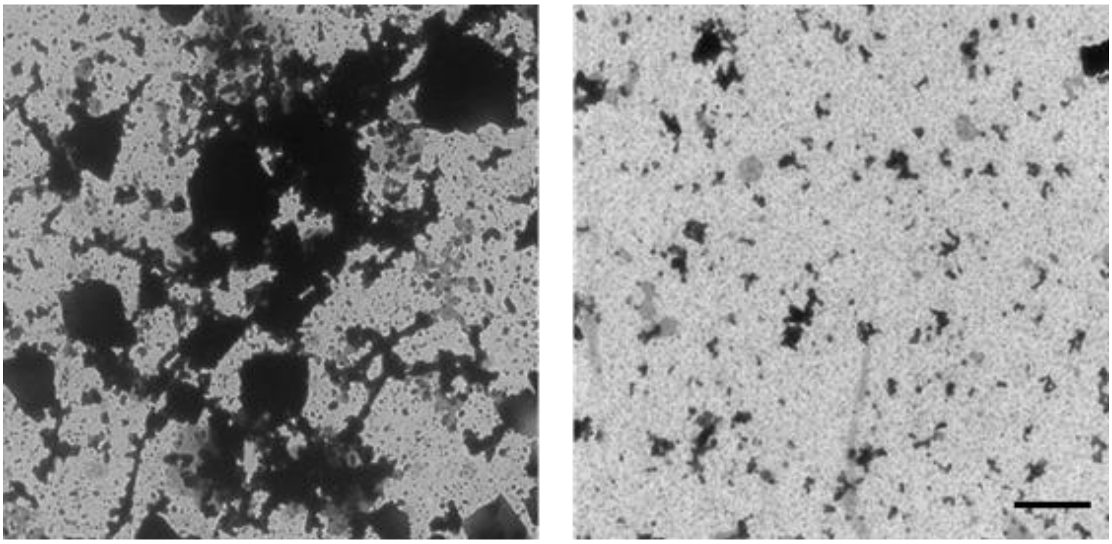

Figure S3. Transmission electron microscopic observation of RA-treatment sample and control. Left. mSAA protein with QD605 incubated for 48 h; Right. mSAA protein with 500 μM RA and 30 nM QD605 incubated for 48 h. Scale bar indicates 1 μm.

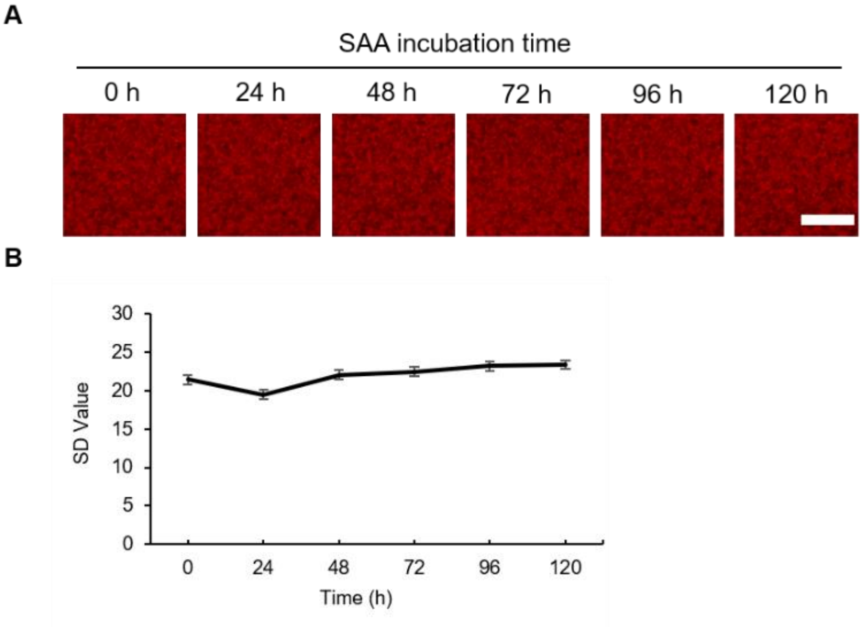

Figure S4. SAA aggregate disruption experiments by RA. (**A**) After incubation of mSAA for 48 h at 37°C, 167 μM RA (final concentration) was added into SAA aggregates, continued to incubate, and observed by a fluorescence microscopy using a 4× objective every 24 h until 120 h. The images show the central area in a well observed by fluorescence microscopy at each time point. Scale bar = 100 μm. (**B**) SD value of each time point as time flow (*n*=3 separate experiments). Each plot represents mean ± SEM.

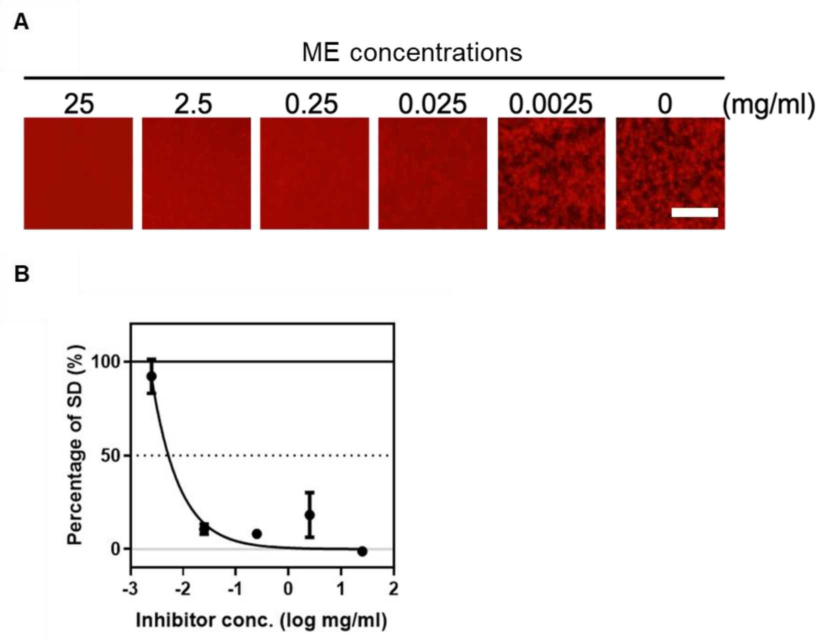

Figure S5. Aβ aggregation inhibitory activity of *Melissa officinalis* extract containing RA. (**A**) Aβ-containing QD nanoprobes were incubated with various concentrations of extracts. (**B**) Estimation of EC_{50} values from inhibition curves (EC_{50} = 0.00525 ± 0.00088 mg/mL) (*n*=3 separate experiments). Scale bar = 100 μm. Each plot represents mean ± SEM.

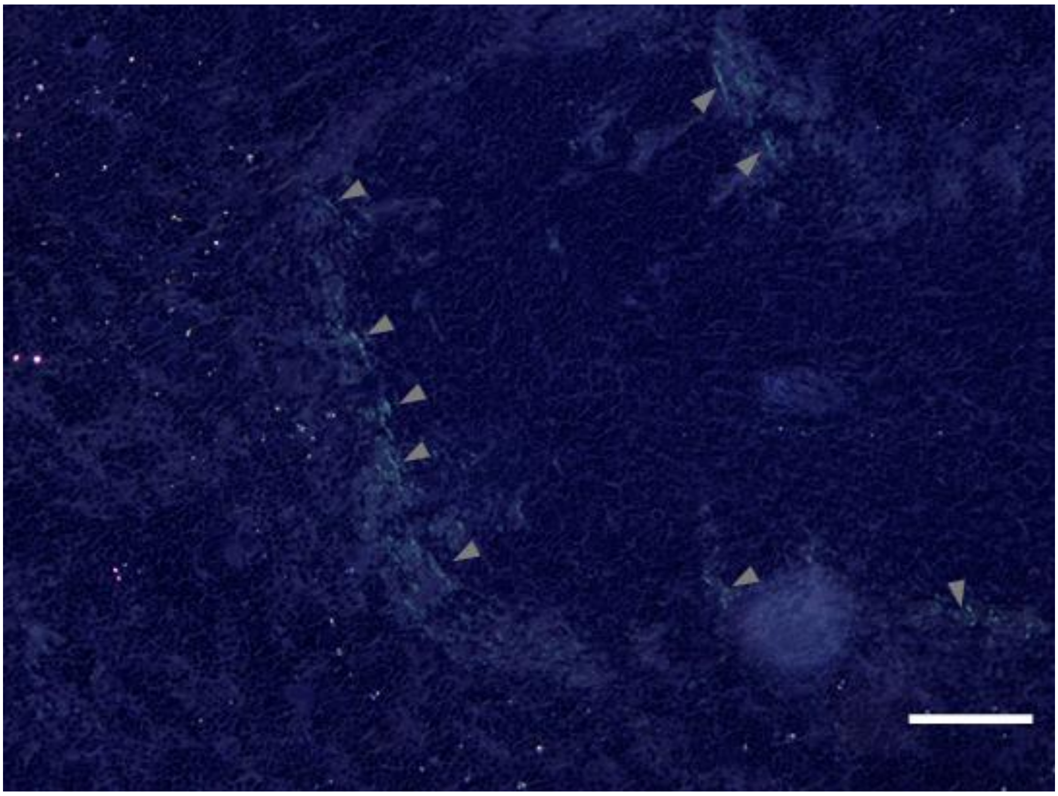

Figure S6. Amyloid deposition in mouse spleen (grey arrowheads). Congo red stain, polarized microscopy. Scale bar indicates 100 μm.

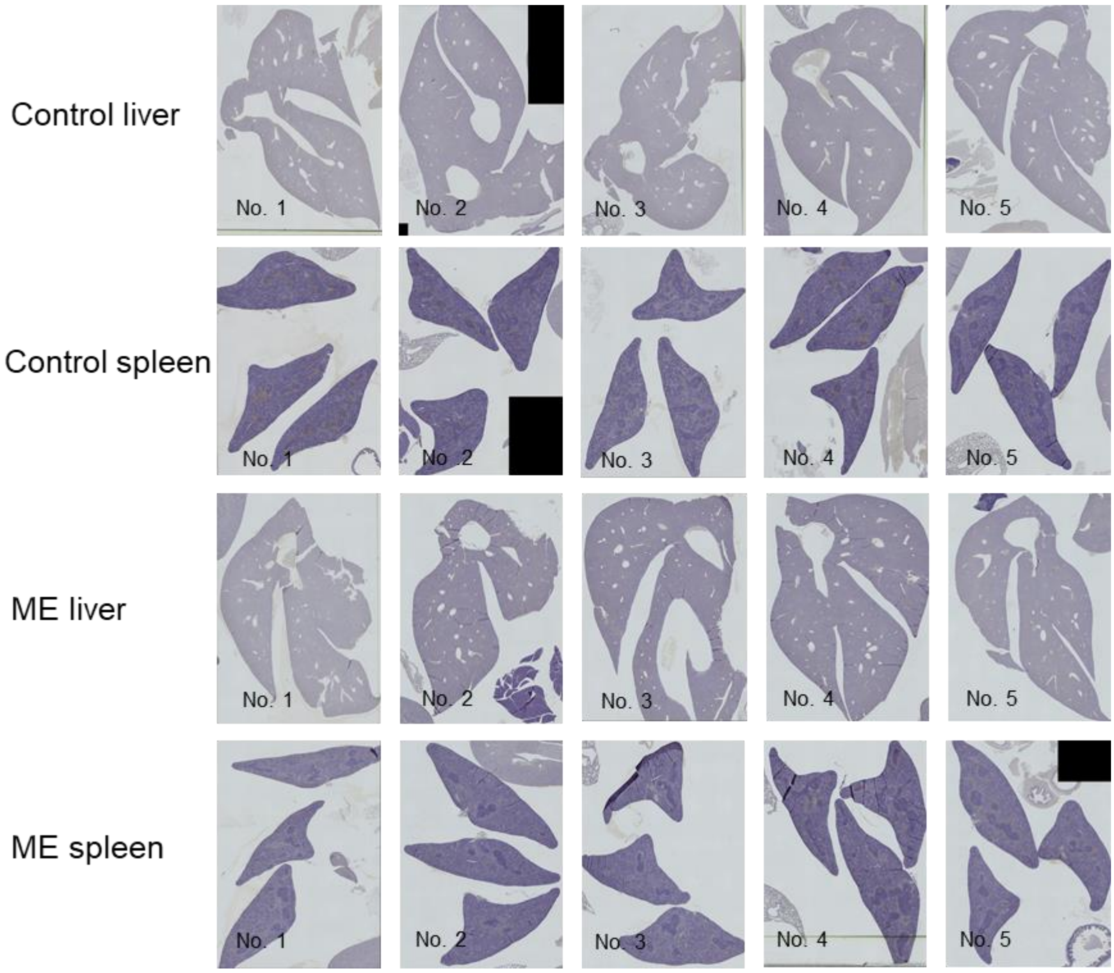

Figure S7. Full imaging of amyloid deposition in mouse organs (liver and spleen) with or without ME Table . Experimentally induced AA amyloidosis detected by IHC staining. In order to better calculate the deposition of amyloid, the black part was not selected when scanning the stitched image of the full organ.
